# Robot initiative in a team learning task increases the rhythm of interaction but not the perceived engagement

**DOI:** 10.3389/fnbot.2014.00005

**Published:** 2014-02-17

**Authors:** Serena Ivaldi, Salvatore M. Anzalone, Woody Rousseau, Olivier Sigaud, Mohamed Chetouani

**Affiliations:** ^1^Sorbonne Université, UPMC Univ Paris 06, UMR 7222, Institut des Systèmes Intelligents et de RobotiqueParis, France; ^2^CNRS, UMR 7222, Institut des Systèmes Intelligents et de RobotiqueParis, France

**Keywords:** social learning, joint attention, engagement, human-robot interaction

## Abstract

We hypothesize that the initiative of a robot during a collaborative task with a human can influence the pace of interaction, the human response to attention cues, and the perceived engagement. We propose an object learning experiment where the human interacts in a natural way with the humanoid iCub. Through a two-phases scenario, the human teaches the robot about the properties of some objects. We compare the effect of the initiator of the task in the teaching phase (human or robot) on the rhythm of the interaction in the verification phase. We measure the reaction time of the human gaze when responding to attention utterances of the robot. Our experiments show that when the robot is the initiator of the learning task, the pace of interaction is higher and the reaction to attention cues faster. Subjective evaluations suggest that the initiating role of the robot, however, does not affect the perceived engagement. Moreover, subjective and third-person evaluations of the interaction task suggest that the attentive mechanism we implemented in the humanoid robot iCub is able to arouse engagement and make the robot's behavior readable.

## 1. Introduction

Personal robots and robotic co-workers need to interact with humans and coordinate with them to fulfill collaborative tasks. Even though a robot has pre-built knowledge, at some point it has to adapt to the way its master (or its human partner) name things and execute the tasks (Wilcox et al., 2012). The robot needs the capability to learn from him as an intelligent social partner (Breazeal, 2003): it must be endowed with tools for learning new skills and symbols, that the human can teach physically and verbally, but also with social skills to interact with humans in a way that is as easy and natural as possible (Huang and Thomaz, 2011; Knoblich et al., 2011). A critical component for natural Human–Robot Interaction (HRI) is the selection and implementation of the social skills that can make the robot “*interactable*,” i.e., usable and understandable also by ordinary people. These skills translate in a combination of verbal and non-verbal communication, which must be adapted in real-time to each human behavior. Clearly, verbal communication is fundamental for information exchange between the partners, to communicate labels, abstract and complex concepts. Non-verbal communication is critical for achieving natural interaction between the human and the robot, especially to achieve engagement and synchronization in tasks where the two are teamed. Implicit non-verbal communication positively impacts human-robot tasks performance (Breazeal et al., 2005).

A possible way to see the team learning problem is to set social parenting scenarios, where a human caregiver supervises the learning process of the robot as an adult would do with a child. The embodiment induced by certain platforms with human-like appearances facilitates the task from the human perspective (Ugur et al., 2011; Natale et al., 2013). This approach is vivid in the developmental robotics community, who made several advancements in the design of theories and mechanisms to make robots learn like children (Smith and Gasser, 2005; Asada et al., 2009). Interestingly, researchers made also several attempts at reproducing the attentive system of infants in robots (Sumioka et al., 2007, 2010), to improve the robot's performances in social learning by regulating contingency and synchrony in the interaction. As in infants (Morales et al., 1998), such systems integrate the social signals exchanged with their caregivers: verbal and non-verbal cues, such as body language, gestures and intentional actions like pointing and gazing. The latter is probably the most important and informative action and the focus of our study.

The gaze is the joint movement of head and eyes used to convey information and indicate situated attention (Staudte and Crocker, 2010, 2011). It is used to indicate interest, intentions and combined with speech it plays an important role in the coordination of joint attention and turn-taking (Meltzoff, 2007; Skantze et al., 2013). It is also used to influence object perception and situated utterance comprehension by driving the interlocutor's focus of attention toward objects of common interest (Moll and Meltzoff, 2011; Obhi and Sebanz, 2011). *Shared attention* or *joint attention* occurs when an agent follows the other partner's gaze to examine an entity of common interest, which is intentionally referred to by the interlocutor through a combination of spoken utterances and non-verbal cues (Emery, 2000; Staudte and Crocker, 2011). Within collaborative actions, joint attention is based on the “shared intentionality” (Tomasello et al., 2005), i.e., the fact that partners coordinate their actions and roles to attain a shared goal, and are mutually aware of the other's perspective (Kaplan and Hafner, 2006). An interesting feature of joint attention is the automatic orientation of attention in response to the perception of the partner focusing on an object or location of common interest (Posner, 1980). Typically, the partner is able to infer the spatial direction of attention on the basis of visual cues (eyes movement, head and body posture). An important characteristic emerging from joint attention is the contingency of the gazing action, and the inter-personal synchrony which is established by the agents when they interact (Delaherche et al., 2012).

Similar behaviors have been observed during HRI, particularly with humanoids. Quoting Chaminade and Okka (2013), “it is known that humans can interpret cues provided by humanoid robots to convey information about their object of attention.” In their study, they showed that a humanoid face can trigger automatic orientation of spatial attention as the human face. Boucher et al. (2012) showed that the robot gaze facilitates the cooperation in a task, making the robot behavior more readable, resulting in faster reaction times. This study puts in evidence that the timing of the attention response during HRI can reveal the human engagement toward the robot and the task. The contingency of robot responses to human cues impacts the humans' behavior in interaction scenarios (Fischer et al., 2013) and is fundamental to establish engagement between the partners (Rich et al., 2010). Particularly in turn-taking scenarios where the partners collaborate in a systematic way, time is critical (Steinfeld et al., 2006): synchrony, delays in replies, and rhythm of interaction can impact on the perception of the robot by the human, amplifying or decreasing the perceived engagement and influencing impression and responsive behaviors. This is true for robots (Thomaz and Chao, 2011) and agents in general (ter Maat et al., 2010). Therefore, there is an apparent link between the timing of interaction, the role of the partners in the interaction and their impressions and responsive behaviors: while this has been shown for virtual conversational agents (ter Maat et al., 2011), there are still open questions about robots in teaching, collaborative and conversational scenarios.

In this paper, we hypothesize that the reaction time of a human in response to a robot utterance can depend on the roles of the human and robotic partners during the interaction. We also hypothesize that the different roles of the partners can influence the rhythm of interaction and the perceived engagement. We speculate that if a human is engaged during the interaction with the robot, and he perceives the interaction as natural as if it was happening with a human partner, then he should exhibit the same features of joint attention as if he was interacting with a human. Particularly, the human should have a rapid gaze response to attention cues from the robot, and react to robot's utterances in a way that is close to the one observed in human–human interactions. Tanenhaus et al. (1995) showed that humans rapidly gaze to objects of interest that are mentioned by a speaker. Does this also happen if the speaker is a humanoid robot? We know from Chaminade and Okka (2013) that the reaction time of the human to the detection of a robot attention cue is longer for humanoid robots than for human faces. Thus we expect the reaction time to robot's attention cues to be globally longer than to human's attention cues. However, we presume that this time can be manipulated by a suitable design of the task (acting on the roles of the partners and their turn-taking), by making the robot more readable and by keeping the human engaged toward the task and the robot.

**Figure 1:**
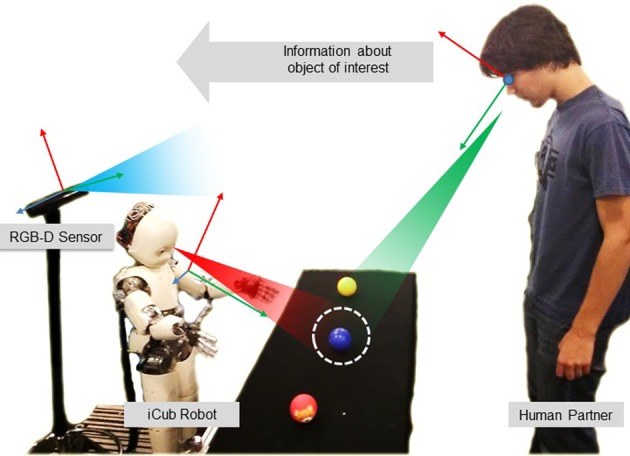
**The object learning experimental scenario**.

We study the effects of a simple joint attention system during a learning task, in terms of induced joint attention (reaction time) and engagement perceived on the human side. We designed a two-step learning experiment, where people had to teach the iCub the color of objects. The learning situation was simple, as shown in Figure 1, and interaction between robot and human was naturalistic. Remarkably, participants were not required to do any calibration nor wear eye-tracking devices, such as in Yu et al. (2010). Objects were selected by simply gazing at them by both pairs, and the information transfer about their color property was based on verbal communication (speech). Human–human interaction studies provide many elements for designing attention mechanisms for robots, based on gaze statistics and observed habits and tendencies. For example Argyle and Cook (1976) report that people look at each other about 60% of the time during an interaction, and look more while listening than talking (during which they give frequent short glances). More fine-grained observations reveal that people look away whenever they start speaking, and that the one finishing a sentence looks at the one who is about to start speaking. Though the implementation of similar mechanisms in the robot would be of interest, for the purpose of our study we designed an attention system that only respects some basic rules of human interactions (Sacks et al., 1974). For example, the fact that only one party talks at a time was ensured by systematically pausing the speech synthesis of the robot after an utterance. This allowed us to define implicitly “roles” and turns in the interaction. We compare in this paper the effect of the initiator of the task in the teaching phase (human or robot) on the reaction time of the human gaze when responding to attention cues of the robot. Through a subjective evaluation, we also assess the difference in the perceived engagement depending on the role of partners during the task. Our guess is that the reaction time could be an index of perceived engagement. Interpersonal coordination is often used to evaluate the attention or engagement between social partners, because it relates to the quality of interaction and cooperation (Wiltermuth and Heath, 2009; Delaherche et al., 2012). Through the subjective evaluation, we seek at finding if differences in the rhythm of interaction can reflect in perceived engagement.

Finally, through a qualitative evaluation by participants to the experiment and observers not involved in the task, we aim to assess whether the simple attention mechanism we implemented in the humanoid robot is able to arouse engagement and make the robot readable.

## 2. Materials and methods

### 2.1. Experimental protocol

**Figure 2:**
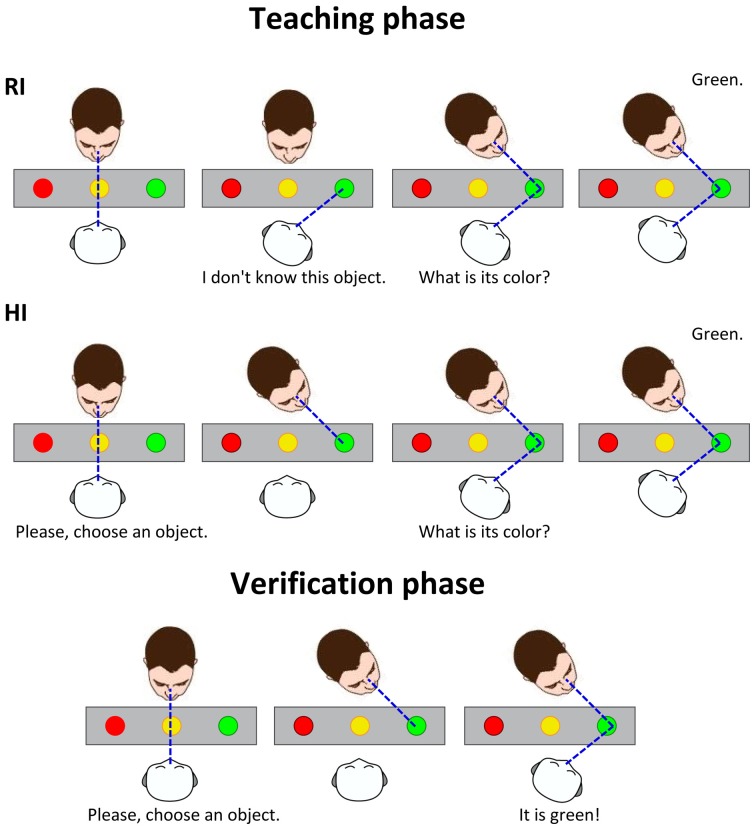
**Schematic representation of the events in the experimental protocol**. Two different conditions are tested for the **teaching phase**. **RI**: The robot initiates the phase by looking at a random object. The human, if engaged, naturally looks back at the common object of interest. When both partners look at the object, the robot asks the color of the object, and the human answers. **HI**: The human initiates the phase by looking at a desired object. The robot tracks the human head movement, then guesses which object is being observed by the human. It asks about the color of the object, which is answered by the human. The color label is then associated to the object's prominent color feature. In the **verification phase**, the human validates if the robot correctly learned the object colors. The human looks randomly at some objects. The robot tracks the human gaze, estimates the object of interest, retrieves the color label learned in the teaching phase and says the color to the human.

The experimental scenario is shown in Figure 1: the robot interacts with a human caregiver to learn the colors of some objects. The human is standing in front of the robot: his/her position is roughly fixed, but not constrained to a specific position with respect to the robot. Between the two, there is a table with several colored objects. When the partners look at the same object of interest (i.e., the blue ball in the figure), they can share knowledge about it. The experiment consists of a supervised learning process, across two phases: a teaching phase and a verification phase. In the first phase, the robot is taught the labels of the objects by the human partner. In the second phase, the human gazes to one of the objects, and the robot responds with the learnt label. The sequences of events in the experimental protocol are shown in Figure 2. Notably, the teaching phase can be performed in two different conditions: *Human Initiative* (HI) and *Robot Initiative* (RI). HI and RI conditions are used to establish which partner initiates the action, that is the first who gazes at the object of interest.

The **teaching phase** is as follows:

(1) The robot waits for the human to establish eye-to-eye contact. When mutual engagement occurs, the robot starts speaking to introduce the next step.

For all the objects on the table:

(2) The gaze leader (HI: human, RI: robot) looks at one of the objects on the table. In the RI condition, the robot simply gazes at one of the objects and asks information about it. In the HI condition, the robot asks first the human to look at an object: this step is necessary to stimulate the human to do the gazing action. In response to the robot request, the human moves his head and eyes to look at one of the items on the table^1^.
(3) The gaze follower (HI: robot, RI: human) looks at the object of interest on the table. In the HI condition, the robot tracks the human head movement, estimating yaw and pitch of the head, then it drives its head to look at the intersection of the gaze direction and the table, which allows the identification of the object of interest. In the RI condition, the robot's head motion is coupled with the information request to the human. This induces joint attention in the human, who naturally looks at the object selected by the robot.
(4) The two partners focus their attention on the same object. The robot asks the human some information about the object (in this scenario its color).
(5) The human tells the robot the object's color. The speech is captured by a lavalier microphone, which improves the quality of the sound source for the speech recognition system. At this point, the human can either look at the robot or at the object (there is no constraint on his gaze).
(6) The robot retrieves the color name from the natural human speech, then it associates the color label to the most prominent color feature of the object observed in the camera image. Once this association is successful, the robot looks back at the human.
(7) The robot greets.

Once the robot has acquired the labels for all the objects, a verification phase begins. In this second phase, the human looks at the objects on the table, one by one in a random order, and the robot says what he learned about this object.

The **verification phase** is as follows:

(1) The robot waits for the human to establish eye-to-eye contact. When mutual engagement occurs, the robot starts speaking to introduce the next step.

Until the human is disengaged:

(2) The robot looks at the human standing in front of it, and asks the human to look at one of the objects on the table.
(3) In response to the robot request, the human moves his head and looks at an object.
(4) The robot looks at the selected object on the table. It tracks the human head movement, estimating yaw and pitch of the head, then drives its head at the intersection of the human gaze direction and the table, which enables to identify the object of interest. The two partners look at the same object.
(5) The robot observes the object in the camera image, and retrieves the color label of its most prominent color feature. Then, it says the color label to the human. At this point, the human is free to gaze at the object or the robot (there is no constraint on his gaze).
(6) The robot greets.

The two phases rely on the induced joint attention mechanism, and on the ability of the robot to engage the human and to be seen as a natural interactive partner. Joint attention is paramount to the success of the two phases: in both phases, the agents must be able to induce joint attention and estimate the gaze of their partner. The readability of the partners is important, as well as their capability to drive the attention of the partner toward the object of interest. Finally, the synchrony between the agents is important to make the pace of interaction as close as possible to a natural one.

We performed the interaction experiment on two groups of volunteers, randomly assigned to conditions HI or RI. The goal was to spot differences in the reaction time of the participants in the two conditions.

After each human-humanoid interaction experiment, we asked the participants to evaluate qualitatively the interaction with the robot through an anonymous questionnaire. The purpose of this evaluation was to verify if the objective evaluation through the attention cues was coherent with the subjective perception.

Finally, we asked external observers, not involved in the experiments, to evaluate the quality of the human–robot interaction. The goal was to obtain an objective evaluation based on the observation of few seconds of human–robot interaction. Indeed, it is possible that the engagement and the quality of interaction perceived by an external observer do not match with the ones perceived by the human interacting with the robot, whose evaluation could be biased by factors related to the task. The observer may notice key elements that make the interaction unnatural or on the contrary very realistic, that may not be obvious to the partner actively interacting with the robot.

#### 2.1.1. Experimental procedure: HRI experiment

We recruited 13 adult volunteers within the local campus population, mostly from the ISIR laboratory, who had no prior experience of interactions with iCub. Volunteers were divided into two groups, and associated to condition HI or RI:

- Group HI consisted of six people (2 males, 4 females), 22 ± 1 years old (age: min. 21, max. 24).
- Group RI consisted of seven people (4 males, 3 females), 26 ± 3 years old (age: min. 22, max. 30).

The only requirement for the participants was the lack of prior experience with the robot; that is we selected volunteers that had never interacted with the robot before^2^. Each participant was involved separately in the experiment, and was not able to communicate with the others before doing the experiment. We informed each volunteer about the teaching task with the iCub through an instruction paper, to make sure that each participant had the same amount of information about the task and the robot's capabilities used during the experiment. Participants were informed about the purpose of the study and the technological limitations of the robot, for example the fact that we could not extract eye movements, so they had to gaze at the objects while trying to keep the eyes fixed^3^. Participants were equipped with a lavalier microphone, and then could enter the room where the robot was waiting for them. They were simply instructed to stay in front of the robot and do the teaching task with the robot, focusing on its gaze. Their position with respect to the robot was not fixed, i.e., they could stay closer or farther. No calibration was required, so once they were in eye contact with the robot, this would speak to start the experiment, in a very natural way. iCub was not moving except for the head. Three colored balls were placed on a table in between the human and the robot, on the left, on the center and on the right. During the two phases, participants were free to speak and interact with the robot in the way they were feeling more comfortable with. The teaching session was composed in both HI and RI conditions of only three trials, corresponding to the three objects to teach. Once the three colors were learnt, the verification phase would begin. This phase was less constrained, in the sense that humans needed to verify that the robot had learnt correctly the three objects, but they were allowed to keep verifying the colors if they wanted to^4^. Once the experiment was over, participants were asked to fill up an anonymous questionnaire to evaluate their experience. There were ten questions evaluating the two-phases interaction through a 1–5 Likert scale.

#### 2.1.2. Experimental procedure: observers

For the evaluation from the observers groups, we recruited mostly volunteers from the local campus population, but also many people from outside the university or research area network. We divided the volunteers into two groups: group A and B.

- Group A consisted of 48 people (79% males, 21% females), 27 ± 9 years old (age: min. 19, max. 62).
- Group B consisted of 34 people (67% males, 32% females), 29 ± 4 years old (age: min. 22, max. 46).

There were no particular requirements for the participants. They were mostly unfamiliar with robotics, and some of them (33% in A, 44% in B) had never even seen iCub before. Participants were directed to a website with the instructions for the anonymous questionnaire, where they were asked to watch a video showing some parts of the human–robot interaction experiment, then answer to a series of questions regarding the video through a web form. Participants in group A would also watch a video where two humans were giving a short demonstration of the interaction task^5^. There were nine questions evaluating the two-phases interaction through a 1–5 Likert scale, then two questions where participants were asked to evaluate the quality of the overall experiment through a first choice selection.

### 2.2. Robotic framework

**Figure 3:**
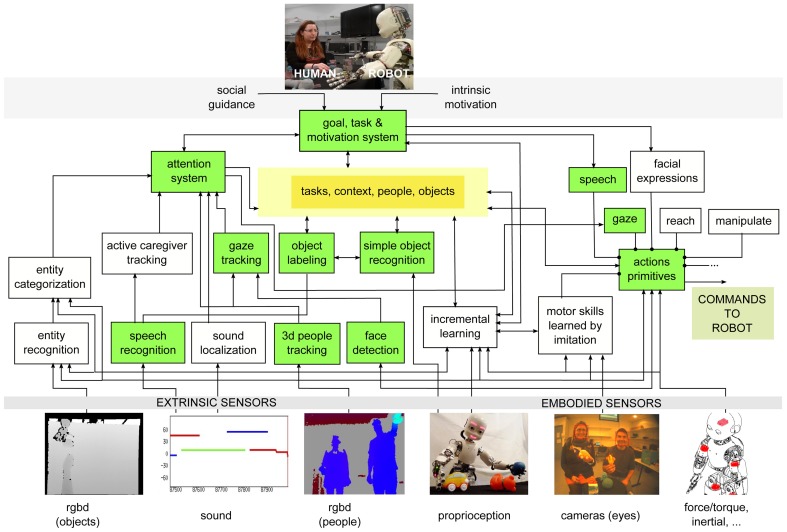
**iCub's cognitive architecture (from Ivaldi et al., 2013) enriched with the new modules**. In green, the modules that have been used for the experiment of this paper.

The robot learns from the human using several coordinated systems (see Figure 3), implemented in its software architecture (Ivaldi et al., 2013). Modules were developed both in YARP and ROS middlewares, connected through a dedicated bridge^6^. A RGB-D camera is placed statically behind the robot, in a convenient fixed position to retrieve human features, whereas the objects are observed through the eyes (i.e., the cameras) of the robot. Verbal communication is achieved through speech synthesis and a simple speech recognition system. The modules are briefly outlined below.

**Figure 4:**
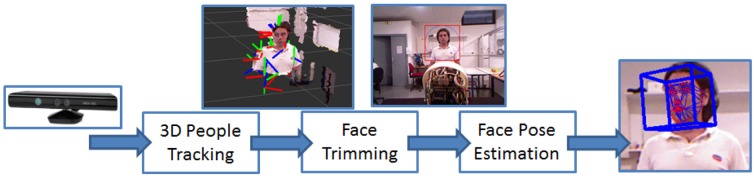
**Sketch of the head pose estimation processing**.

- *3D People tracking*: we use a multiple skeleton tracking system by OpenNI to track the position of humans (Shotton et al., 2011) through the information perceived by the RGB-D sensor. After a background subtraction step applied to the depth image in which the static environment is separated by the moving bodies, the points related to the human figures are analyzed and classified according to depth invariants and 3D translation invariant features. By this classification, the system will be able to estimate which part of the body each depth pixel belongs to. From these classified patches, the position of each joint is calculated according to their density.
- *Head Pose estimation*: the 3D position and orientation of the human head is retrieved by fusing the information from the skeleton tracker with the information retrieved by the color image perceived by the RGB-D sensor. More in detail, the 3D head position is projected on the 2D color image. As shown in Figure 4, the latter is cropped accordingly in order to obtain an image in which the face is found; then the Constrained Local Models algorithm (Cristinacce and Cootes, 2006), a standard face pose estimation and tracking algorithm, is applied and the head pose is extracted.
- *Gaze tracking*: the gaze of the human partner is approximated by his head orientation (pitch and yaw). The estimation is thus inaccurate, but it is the simplest way that does not necessitate the use of external devices (e.g., eye trackers) or the availability of high-resolution cameras to observe head and eye movements.The adopted solution is also non “invasive,” resulting in a more natural interaction. More details on this module can be found in Rousseau et al. (2013).
- *Simple Object Recognition*: a simple object recognition module assumes the object of interest to be roughly at the center of the visual field of the cameras. This is consistent with the fact that the robot gazes at the objects pointed out by the human partner, so the object is centered in the camera images. A simple image processing pipeline is used to segment the object from the background, extract its contours and compute its most characteristic color, which is used for label association.
- *Verbal communication*: the robot communicates through a text-to-speech synthesis module based on Festival, and a speech recognition module based on CMU Sphinx^7^.
- *Object labeling*: we defined a simple grammar to identify the color label from the natural speech of the human partner interacting with the robot^8^. The label is then associated to the colorimetry feature of the object, identified by the object recognition module from the camera images^9^.
- *Robot motion*: the robot produces smooth human-like movements using a minimum-jerk Cartesian controller (Pattacini et al., 2010; Ivaldi et al., 2012). It must be noted that we constrained the robot's movements, so that the head was the only moving robot part (neck-3DOF and eyes-3DOF are actuated). We intentionally did not make the robot move its trunk or its arms, for example for pointing the objects, because we wanted to avoid the human gaze to be perturbed by proactive gaze effects due to robot gestures (Sciutti et al., 2012; Flanagan et al., 2013).

#### 2.2.1. Performances

The overall performances of the robotic software system depend on the reliability of several interconnected modules. In preliminary tests (Rousseau et al., 2013), we evaluated the performance of each component: the object recognition system was correct in 92% of trials, while the speech recognition system had only 76% of correct labels. This latter result may be negatively influenced by the uncalibrated speech model and the different mother languages of the participants to the experiments. The gaze tracking system, based on the estimation of the head pitch and yaw, was evaluated through comparison with a motion capture system. The positive match was 49% for the pitch and 93% for the yaw. The error brought by the pitch evaluation was compensated in the experiments by a suitable placing of the objects on the table, so that the robot could easily discriminate the human vertical gaze direction. We are aware that this framework is less precise than others in the evaluation of gaze (we also cannot easily include eyes movement tracking), however, it provides an online estimation/approximation of the human gaze in natural HRI conditions. It can be used as a feedback signal to adapt or modulate the behavior of the robot in real-time HRI experiments.

#### 2.2.2. Measurements

**Figure 5:**
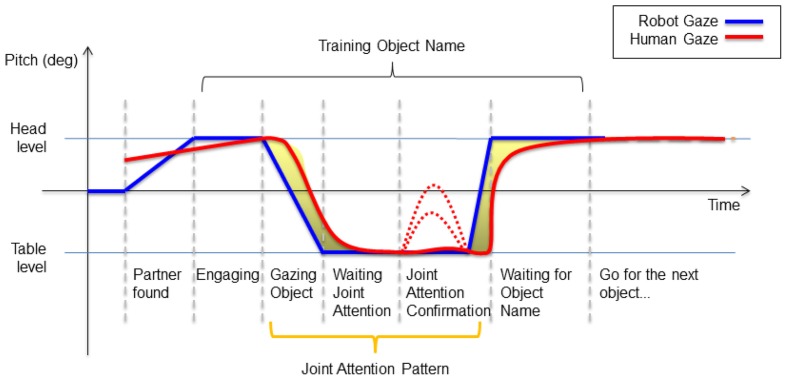
**Ideal timeline for the human's and robot's gaze during the teaching phase of the experiment, in the RI condition**. The robot gaze (blue line) is moved toward the human head to look for eye contact. When mutual eye contact is established, in the RI condition the robot lowers the head to look at one of the objects on the table. The human, if engaged, follows the robot gaze (red line). When both partners look at the same object, the information exchange can take place. In this situation, the human can keep gazing at the object or look back at the robot for a confirmation (dashed lines).

**Figure 6:**
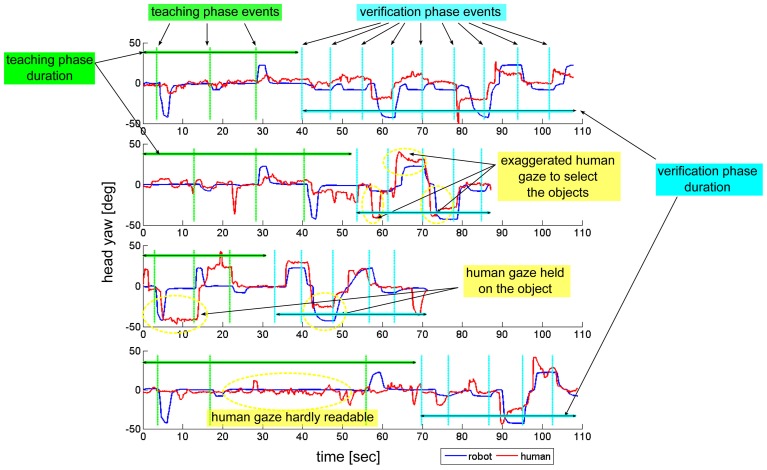
**Some examples of the humans' and robot's gaze during the experiments**. Four participants from the RI condition are shown. Blue lines correspond to the robot gaze, while red lines correspond to the human gaze. The head yaw is shown, so there are three visible locations corresponding to the three objects on the table. In the first part of each plot, the green vertical lines mark the beginning of each phase where the robot starts looking at an object of interest. In the second part of each plot, the cyan vertical lines mark the beginning of each phase where the human looks at an object of interest.

The course of the experiment is controlled by a finite state machine. The timing of the events generated by the computer, the robot actions and the participants' responses are imported from the log of each experiment. The human gaze is continuously estimated from the RGB-D flow through the gaze tracking module. Figure 5 is an example of the ideal timeline of the robot's and human gaze that we were expecting to observe during the learning phase of the RI experiments^10^. The *y*-axis is the head pitch, marking a high-level for the head and a low-level for the table where objects are located. The *x*-axis is time. In the first part of the plot the robot gaze (blue line) and the human gaze (red line) met at some point, as to establish eye-to-eye contact. When partners are engaged, the robot lowers its head to look at an object of interest: the robot gaze moves to the table level. If the human is engaged, his gaze will follow the robot and they will both look at the same object of interest. When the robot has successfully retrieved the object's information from the human, its gaze goes back to the head level, i.e., the robot looks at the human face. It must be noted that the robot asks the question about the object only once it has verified that the human is correctly gazing at the object of interest, so the human is “forced” to turn its head in a way that is sufficiently explicit for the robot to estimate his gaze correctly. At this point, the human can have different gaze responses: he can hold his gaze on the object, he can look back at the robot and stare at the robot as if he was expecting an acknowledgment, he can quickly look at the robot then again at the object, and so on. We observed different gaze behaviors in this phase. Some examples are shown in Figure 6,^11^: in the first plot, the human quickly looked at the objects then came back looking at the robot; in the second, the human exaggerated the movements of his head to be more readable by the robot; in the third, the human held his gaze on the objects until he was sure that the robot had learnt its color; the fourth plot shows some hesitations of the humans, that made his gaze hardly readable by the robot. Overall, we observed different behaviors in the participants. Since they had never interacted with the robot before, any difference in their gaze strategy is to be attributed to individual differences, that are not relevant to our study.

## 3. Results

### 3.1. Attention analysis

The effect of the two conditions is reflected in the gaze response of the participants in the verification phase. We retrieved two important measurements. The first is the reaction time of the human in response to the attention stimulus of the robot, i.e., the request to select an object. We measure in this case the time elapsed between the onset of robot speech and the time when the human gaze, stabilized on the object of interest, is correctly identified by the robot. The second is the interval between two successive requests from the robot, marking the amount of time dedicated by the partners to exchange information about the object of interest. This measurement is inversely proportional to the *pace of interaction* as it has been defined by Rich et al. (2010). The shorter the interval, the higher the pace or the faster the rhythm.

**Figure 7:**
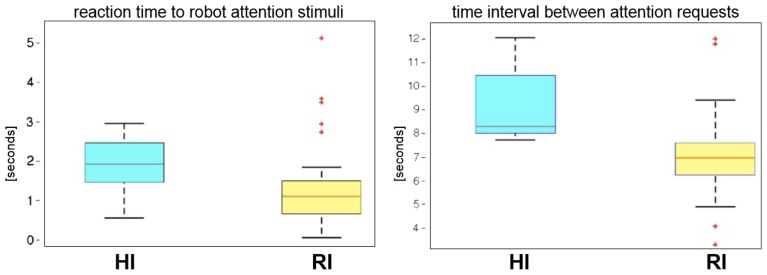
**Reaction time to robot attention stimuli and time interval between consecutive attention requests in the verification phase**.

Tables 1, 2 report the reaction time and the indirect pace measurement for the participants of the two groups. The time distributions were compared with Wilcoxon's test. The test shows that there is a difference in the timing between the two groups (*p* ≤ 0.005). People in the RI group react faster than the ones of the HI group, and the interaction with the robot has a higher rhythm (see Figure 7).

**Table 1:** **Reaction time (seconds) in response to robot attention stimuli (utterances) during verification phase**.

**Group**	**Mean**	**Std**	**Median**	**Wilcoxon's test**
HI	1.932	0.711	1.917	W = 418,
RI	1.296	1.145	1.106	*p*-value = 0.005

**Table 2:** **Time interval (seconds) between consecutive robot attention stimuli (utterances) during verification phase**.

**Group**	**Mean**	**Std**	**Median**	**Wilcoxon's test**
HI	9.524	1.515	8.588	W = 447,
RI	7.287	1.653	7.257	*p*-value = 1.6e–5

**Figure 8:**
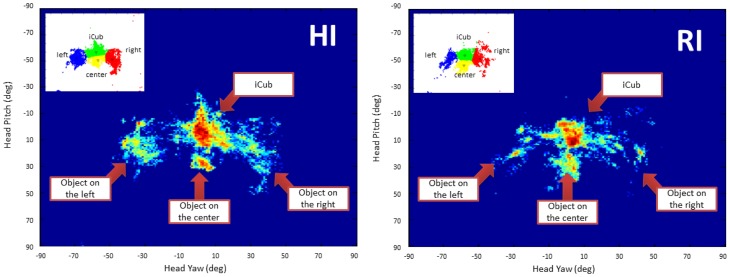
**Normalized gaze heat map of the human partners in the HI and RI groups**. The plots show the points in the pitch-yaw space representing the gaze direction of the human partners during the interaction with the robot. Note that the range of pitch and yaw is [-90°,90°]. For the head pitch, −90 is on top of the head, 0 is in front of the head, 90 is below the head. For the yaw, 0 is in front of the head, while −90 and 90 represent **left** and **right**.

Figure 8 shows the normalized gaze heat-maps of the two groups. Each map is a plot in the head's pitch-yaw space, thus each point represents the gaze direction of the human during the interaction with the robot. The range of pitch and yaw is [−90°,90°]. For the head pitch, 90 is on top of the head, 0 is in front of the head, −90 is below the head. For the yaw, 0 is in front of the head, while −90 and 90 represent left and right. We were able to identify the four clusters associated to the robot head and the three objects by applying K-means on the points, indicated in the left upper corner of each plot. The mean and standard deviation of the centroids of the four clusters is reported in Table 3. We compared the density of each cluster in the two conditions with Wilcoxon's test. The test showed that there is no significant statistical difference in the clusters for both conditions (*p* > 0.1). It is, however, interesting to observe the amount of time spent by the participants in looking at the different salient topics, which is proportional to the density of the clusters. Overall humans spent 66% of their time looking at the robot. For the three objects, the amount of time is unequal: while the left and right objects get almost the same amount of time (7% and 6%), the object in the center was the focus of attention for almost twice the time spent for the others (21%). This has a double explanation: on one side, it is more difficult for the robot to detect that the human has moved the head to gaze at the object of interest if the movement is exclusively on the head pitch; on the other side, sometimes participants spontaneously looked downward to match the robot's gaze (this behavior is in fact “normal” for humans and rather a positive sign of natural, engaged interaction with the robot).

**Table 3:** **Characteristics of the four clusters of gaze points, corresponding to robot and objects, in the normalized head's pitch-yaw space of the participants in HI and RI groups**.

**Target**	**Phase**	**Yaw mean**	**Var**	**Pitch mean**	**Var**	**Density mean**	**Std**	**Wilcoxon's test**
Robot	HI	2.51	31.52	0.40	42.38	0.647	0.197	W = 22,
	RI	2.24	26.05	5.61	31.31	0.673	0.259	*p*-value = 0.804
Left object	HI	−33.56	50.08	13.92	172.13	0.089	0.050	W = 37,
	RI	−25.45	47.44	13.54	109.67	0.052	0.049	*p*-value = 0.128
Center object	HI	5.88	34.21	18.78	59.25	0.190	0.213	W = 26,
	RI	0.82	20.96	28.08	39.05	0.229	0.258	*p*-value = 0.901
Right object	HI	31.60	51.44	16.94	176.88	0.072	0.054	W = 32,
	RI	29.58	85.28	12.73	118.95	0.045	0.037	*p*-value = 0.383

*Mean and variance refer to the coordinates of the x- and y-axis (yaw and pitch) of the points in each cluster. Wilcoxon's test was applied on the normalized densities of each cluster*.

### 3.2. Subjective evaluation

Table 4 reports statistics about the evaluation of the volunteers involved in the object learning task. Overall, participants perceived the robot as quite engaged (nobody evaluated engagement with negative marks—i.e., below 3); the robot behavior was quite readable, as participants pointed out that it could understand the task and it was easy for them to figure out when the robot was expecting an input from the human. The gaze behavior of the robot was also quite explicit, but this seems quite obvious because the objects position was facilitating the task. Interaction with the robot was globally easy for the subjects, despite the technical difficulties in making the interaction robust with respect to the participants subjectivities. The task performances, indeed, were influenced by the irregularities introduced by each participant: some had difficulties in having their speech recognized correctly (we did not calibrate the speech recognition system for the individual participants), some had a very stiff pose and their head was not turning to look at the objects, etc. It is likely that these variables influenced negatively some subjects, which could explain the fluctuation of some evaluations. Clearly, if subjects agreed on the ease/naturalness of interaction, they were uncertain about the human-likeness of this interaction. This result is not surprising, because we constrained the interaction to be based simply on gaze. Even if we asked the subjects to concentrate on the gaze, it is possible that, to feel it “human-like,” they would have needed something more, for example more body language. It is also possible that the evaluation of the interaction by the subjects may be influenced by other factors: first, the surprise effect due to the first encounter with a humanoid robot, which was unknown to most of them; second, the frustration of not having successfully completed the task for factors not correlated directly to gaze (for example speech). Finally, the judgment of the “human-likeness” of the robot may also be influenced by the expectation the subjects had during the interaction. During the task, their behavior unconsciously adapted to the robotic partner.

**Table 4:** **Post-experiment questionnaire for participants**.

**Question**	**Group**	**Mean**	**Std**	**Min**	**Max**	**Wilcoxon's test**
Q1: The robot was engaged during the color naming task	HI	4.50	0.84	3	5	W = 28.5, *p* = 0.2824
	RI	4.00	0.82	3	5	
Q2: The robot understood the task	HI	3.83	1.47	2	5	W = 19.5, *p* = 0.8795
	RI	4.14	0.89	3	5	
Q3: The robot could be a good partner in a cooperative task with a human	HI	2.33	0.52	2	3	W = 12, *p* = 0.1767
	RI	3.00	1.00	2	5	
Q4: The robot is intelligent, it understands what happens	HI	2.33	0.52	2	3	W = 15, *p* = 0.4028
	RI	2.86	1.21	1	4	
Q5: During the naming task, robot was showing a human-like behavior	HI	2.17	0.75	1	3	W = 18.5, *p* = 0.7428
	RI	2.43	0.79	2	4	
Q6: During the task, the robot behaved like a child	HI	3.17	0.76	2	4	W = 31, *p* = 0.1572
	RI	2.29	1.11	1	4	
Q7: Interaction with the robot was easy	HI	3.33	1.21	2	5	W = 18.5, *p* = 0.7682
	RI	3.57	1.13	2	5	
Q8: The robot understands which object is indicated by the human	HI	3.50	1.22	1	5	W = 26, *p* = 0.4811
	RI	2.71	1.60	1	5	
Q9: It was easy to identify the object indicated by the robot	HI	4.33	0.82	3	5	W = 15.5, *p* = 0.4069
	RI	4.71	0.49	4	5	
Q10: It was easy to see when the robot is waiting for something from the human	HI	4.00	1.26	2	5	W = 18.5, *p* = 0.7542
	RI	4.29	1.11	2	5	

### 3.3. Evaluation by third-persons

Table 5 reports the statistics about the first part of the post-experiment questionnaire given to external observers (mean, standard deviation, minimum and maximum value assigned to evaluate each question). Statistical analysis was realized in R through the Welch two-sample *t*-test, comparing the answers of all questions for the two groups. Significance level was set at 95%. The analysis shows that there is no significant difference between the A and B conditions: for each question *p* > 0.4, which indicates that the vision of the human–human interaction demonstrating the task did not influence the perception of the observers. This means that seeing a human–human demonstration of the task is not a bias in judging the human–robot interaction task. Overall, participants evaluated positively the aspects related to the task (Questions 2, 7 especially). The “human-likeness” of the gaze and more generally the robot's behavior is above the neutrality threshold, so rather positive. This result can be explained by looking at the second part of the questionnaire, where the participant had to indicate the main issue of the teaching phase of the experiment as well as the most important feature that in their opinion would have been necessary for a more human-like interaction. Participants could choose among a list of predefined items, but could also propose their own. Table 6 reports the percentages of answers for each group. For both groups, two are the main issues with the teaching phase of the experiment. The first is the “slowness” of the robot that replies with a certain delay to the human labeling; it also keeps the pace of interaction globally low with respect to what a human would do. This is especially remarked by the participants of group A, who saw the human demonstration (that was quicker than any human–robot interactions shown in the video). The second negative point is about the robot's movements that are not perceived as natural enough. This can be explained by looking at the answers of Question 11, where participants could indicate the critical features to add to make the robot more “human-like.” In Question 11, almost half of the suggestions were indicating faster movements, which is coherent with the first issue pointed out in Question 10. Almost one third of the participants, in both groups, suggested features linked to gestures and movements to make the robot more readable (e.g., give more feedback to the user). It may seem straightforward, but pointing at the objects while gazing at them would certainly make the robot appear more natural and “animated” to the partner's eye. In the experiments we constrained the robot movements to the head, because we did not want to have the human gaze perturbed by proactive gaze, such as the one that could be induced by the human following the hand and predicting the target pointing location before the robot would gaze at it. This choice would allow us to have a better estimation of the human gaze at the cost of a less natural robot's behavior. Interestingly, this lack of “naturalness” was noted by the external observers but not by the human partners during the experiment. Among the desirable features that could make the robot more “human-like,” participants in both groups indicated speech. In our experiments, the robot's speech is the default voice of a classical text-to-speech tool (Festival). The speech is cold, monotone: perfect for a robot, and nothing like a human's. Probably, external observers would prefer a more human-like voice, with the ability to change the pitch or the intonation to give further feedback to the partner or the observer himself (for example: communicate enthusiasm when the robot learns something new).

**Table 5:** **Post-experiment questionnaire for external observers**.

**Question**	**Case**	**Mean**	**Std**	**Min**	**Max**	**Welch *t*-test**
Q1: The robot was engaged during the color naming task	A	4.18	0.87	2	5	*t* = 0.325; *p* = 0.74
	B	4.08	1.05	1	5	
Q2: The robot understood the naming task	A	4.33	0.83	1	5	*t* = 0.076; *p* = 0.93
	B	4.38	0.95	1	5	
Q3: The robot could be a good partner in a cooperative task with a human	A	3.25	0.96	1	5	*t* = 0.022; *p* = 0.98
	B	3.29	1.12	1	5	
Q4: The robot is intelligent, it understands what happens and learns something	A	3.48	1.20	1	5	*t* = −0.825; *p* = 0.41
	B	3.62	1.07	1	5	
Q5: Interaction with the robot is easy	A	3.31	0.97	1	5	*t* = −0.093; *p* = 0.93
	B	3.32	1.03	1	5	
Q6: During the naming task, the robot was showing a human-like behavior	A	3.06	1.02	1	5	*t* = 0.388; *p* = 0.70
	B	2.97	0.99	1	5	
Q7: The robot understands which object is indicated by the human	A	4.5	0.74	1	5	*t* = 0.479; *p* = 0.63
	B	4.32	0.98	1	5	
Q8: The robot gaze is human-like	A	3.29	1.11	1	5	*t* = 0.272; *p* = 0.79
	B	3.32	1.03	1	5	
Q9: It was easy to see when the robot is waiting for something from the human	A	3.69	0.93	2	5	*t* = 0.063; *p* = 0.95
	B	3.62	1.07	1	5	

*First part (grading behaviors)*.

**Table 6:** **Post-experiment questionnaire for external observers**.

**Answer**	**A (%)**	**B (%)**
**Q10: In your opinion, what is the main problem in the teaching phase?**		
The robot movements are not natural	27.08	29.41
The robot is slow	52.08	26.47
Not natural speech interaction	2.08	−
The robot does not understand the human speech	2.08	−
The human is slow	4.17	2.94
Difficult to understand what the robot wants to do	4.17	11.76
There are no problems in this phase	8.30	14.71
Human movements are not natural	−	5.88
The learning task is not natural	−	2.94
**Q11: In your opinion, which of these features will make the robot more human-like?**		
A faster interaction	45.83	35.29
A better voice, more natural	18.75	26.47
The robot making gestures	22.92	23.53
The robot speaking with a different vocabulary	2.08	−
Body language (incl. walking)	4.17	−
The robot giving more feedback to the human	6.25	14.71

*Second part (“first choice” selection)*.

## 4. Discussion

### 4.1. Does an active robot induce a faster interaction?

The response times reported in Table 1 show that humans respond faster to robot's utterances in the verification phase when in the previous phase of the task the robot was leading the interaction (RI condition). The measurements verify our initial hypothesis, that is the difference in the initiator/leader of the learning task in the first phase is reflected in different reaction times in the second phase of the task. In the HI teaching phase, the robot asks the human to choose an object, leaving the choice to the human, and making him the main actor of the interaction. Once the human has gazed to the object, and its gaze is correctly estimated, the robot looks at the object of interest. The rhythm of the interaction is essentially determined by the human response to the robot's utterance: in terms of time, the human can move more or less quickly his head, and make the movement more or less “readable” by the robot, thus influencing the time needed by the robot to estimate the head direction correctly. Once the direction is estimated, the robot moves its eyes and head with a practically constant movement, determined by the gaze controller—the same used in Boucher et al. (2012). In the RI teaching phase, the robot randomly picks an object on the table and asks the human to tell the color of the object. The choice in this case is made by the robot, which initiates the interaction. The rhythm of the interaction as well as its success is determined by the readability of the robot, its capability to induce in the human a prompt response to the robot attention request, and of course by the readability of the human that needs to have the same referential focus as the robot to make the interaction advance^12^. Again, the duration of the robot's movements is fixed, so the human is the main actor responsible for setting the pace of the interaction through his behavior. Why do these two conditions reflect in different reaction times in the verification phase? There could be several reasons. One possible reason is that in the RI condition, participants learned how to “read” the robot behavior to advance in the teaching phase, and reply to its questions contingently. Therefore, in the verification phase they could be facilitated in responding promptly to the robot attention request. Another possibility is that, in the RI condition, the robot is interacting in a more “active” way, because it asks questions about the objects. As observed by Breazeal (2003), this pro-active behavior regulates the interaction and provides a feedback signal to the human about the internal state of the robot. This behavior is also likely to induce in humans a social parenting effect: humans could have the impression that they are teaching the objects properties to a curious child. Conversely, in the HI case the robot acts “passively”: it asks the human to provide the attention stimulus. So not only the learning process is led by the human, but the human could also be more hesitating in front of such request. The active/passive attitude could be responsible for making the robot more transparent to the human, in a way that the human would have or not a clear intuition about the robot's internal state. This claim is partially supported by some negative evaluations provided by the participants in the post-experiment questionnaire (see Table 4), such as Questions 3–5. To summarize, the prior experience of an “active” robot leading the learning task makes the human react faster to the robot's attention utterances. Among the possible reasons, the robot active attitude improves its readability and the intuition of the human about the robot's state, hence the human reacts faster when he is interrogated by the robot. Our observations can be put in relation with the ones of Huang and Thomaz (2011), where they showed that “a robot responding to joint attention is more transparent, such that interactive task performance is faster and more efficient.”

### 4.2. Functional roles and social tasks

The learning experiment described in this paper is rather simple, yet it addresses an up-to-date issue in the domain of human–robot cooperation, regarding functional roles of partners during interactions. Roles can be described in terms of behaviors, rights, expectations and norms that humans follow during social interactions. We speculated that the different roles of the robot in the task (initiator vs. follower) could have an impact on the pace of the interaction. In the two conditions proposed in the experiment, the robot initiative determined its attitude as a “leader” of the interaction or as a “receiver.” Leaders (or “givers”) express proactive behaviors, stimulate the partner and generally set the time in turn taking scenarios. On the other hand, “receivers” follow the leaders' initiative by responding to the stimuli proposed by them. In the RI condition, the robot was leading the interaction by actively choosing the object of interest and asking questions to the human, but especially setting the onset of each turn. The HI condition, the robot was marking the beginning of each turn, by asking the human to choose an object, but in fact the pace was determined by the human reaction time to the robot request and the human decision about where to gaze, when and how. It is clear that the pro-active element in the two conditions was decisive for setting the rhythm of the interaction in the two phases. The remarkable result of our study is that it also influenced the rhythm of the verification phase. Though preliminary, our study suggests that the leading role of the robot can influence the pace of the interaction in a social task. The more general question is whether different functional roles (i.e., teacher vs. learner or requester vs. giver for example) can have impact on the dynamics and performance of the interaction.

During social tasks, people rely on their ability to share representations, predict the partner's actions and intentions, integrate the other's actions and the predicted effects; they need a constant verification about the mutual understanding and the “mutual awareness” of the partners across the evolution of the interaction (Sebanz et al., 2006). As discussed in section 4.1, in our experiment the robot's pro-activity makes its behavior more understandable, as it provides an additional element to the human to estimate the “internal status” of the robot and be aware of the progression of their interaction. The same happens in human–human interaction, where mentalization and meta-cognition are entailed to take account of the knowledge and intentions of others (Frith, 2012). This is well explained in Clark's theory of human–human collaboration (Clark, 1996): people enter in joint activities when they have to solve coordination problems, and to achieve coordination partners need to constantly update their common ground via the available methods of communication (verbal or non-verbal). Tomasello (2009) identifies in coordination and communication the primary processes for establishing cooperation. Role theory provides further arguments to explain this behaviors from a sociological point of view, based on the premise that persons in social positions hold expectations for their own behaviors and those of others: according to Biddle (1986), humans behave differently and in a way that can be predicted depending on their respective social identity and the situation. Role taking has been also studied in the context of social and infant development for cognition (Piaget, 1962). In a study of verbal communication during a collaborative task, Clark and Wilkes-Gibbs (1986) modeled the acceptance as a step by step process, started by a “initiator,” where the partners establish the mutual belief based on the evidence of common references.

The modality of interaction, the relationship between the human pairs, has also influence on the action patterns at kinematic level: Georgiou et al. (2007) showed for example that cooperative and competitive behaviors translate into different kinematic trajectories for reach-to-grasp tasks in human–human interaction. The definition of roles and the timing of their actions are critical. Synchronization in particular depends on “readability” of the partners, the way humans detect and read non-verbal cues, the ability to anticipate the reactions or the intent of the human partner and enter into synchrony. The idea is simple: the less feedback you have from the partner (or the more this feedback is ambiguous), the more time the task will take. In humans, the study of synchronization skills has fundamental applications in the early diagnosis of social disorders as autism (Delaherche et al., 2013). Regarding synchrony and temporization, Yamazaki et al. (2008) analyzed human–human interaction to highlight some transition relevant places (TRPs) corresponding to the moments when a speaker's turn is about to end. Emphasized keywords, unfamiliar and deictic words are other examples of focus points. When placing the non-verbal actions at these points, the participants of the interaction perform more non-verbal actions with a precision timing.

In robotics, for the design of social agents that can enter into synchrony with the partner (Andry et al., 2011), for the design of robot controllers that adapt to the human decisions during collaborative tasks (Wilcox et al., 2012). As discussed by Stefanov et al. (2009), however, “there are only a few studies discussing roles in human–human interaction,” and most of them are focused on verbal communication (e.g., the dominant partner in a conversation Hung et al., 2008) whereas it would be more interesting for robotics to explore the impact of such roles for tasks where non-verbal cues are predominant, such as joint attention and haptic human–human interaction (Reed et al., 2007). An interesting review of roles assignment policies for human–robot motor interaction can be found in Jarrassé et al. (2013).

To summarize, there is evidence that verbal and non-verbal behaviors, interplays and interpersonal synchrony are critical to determine the functional role of humans during social tasks (Dong et al., 2013). However, while the literature in psychology analyzing the social and cognitive aspects of roles is notable, there are only few studies describing the impact of roles into the dynamics of interaction.

Since interpersonal synchrony can be used as a measure of engagement between social partners (Wiltermuth and Heath, 2009; Rich et al., 2010; Delaherche et al., 2012), from the results presented in section 3 we can contend that the leader role of the robot increases the rhythm of interaction for our social learning task. Whether this statement can be generalized to other tasks or to different roles, it is an open question. Surely more experiments are needed to elucidate this question.

### 4.3. Readability and gaze estimation

Humans are extraordinarily capable of inferring with accuracy the spatial direction of attention of their partners. They can integrate cues from the partner's posture, eyes, and anticipatory signals (Sumioka et al., 2007) as well as linguistic information (Tanenhaus et al., 1995). If the robot is sufficiently “readable,” i.e., provides cues about its intentions or manifest them explicitly, the human would have no difficulty in inferring its focus of attention. In our experiment, we made the robot readable with a simple combination of gaze and speech, which is known from literature to be sufficient for accomplishing the proposed learning task (Staudte and Crocker, 2010). This fact was confirmed by our experiments: subjects interacting with the robot indicated that it was easy to identify the object indicated by the robot (see Table 4). Nevertheless, participants also pointed out the necessity of adding more gestures and making movements more natural, which suggests that our attention system was not sufficient for a “human-like” interaction. It is likely that the constraints we put on the robot's body were the main cause: adding the torso motion (for example a swing toward the left/right), pointing actions and gestures, would have certainly made the robot movements fancier and more natural—that is closer to the ones a human naturally makes when showing objects to another partner. Indeed if the robot has arms and torso, why is it not moving them? We already explained that our choice was motivated by the need to avoid proactive gaze in the human, but neither the naive participants performing the experiment or the external observers were informed about this issue. However, it is very likely that the main constraint that perturbed the participants was in the attention system in first place. When two people interact in face-to-face scenarios, their eyes/heads are constantly moving, following the referential focus of the partner. Realizing such joint attention mechanism in the context of human–robot interaction is technically challenging, because of the difficulty in estimating the human referential focus from the robot's cameras. Researchers often relies on external wearable devices, eye-trackers (Yu et al., 2010), or simplified object pointers to select explicitly the target of joint attention (Huang and Thomaz, 2011). Those solutions either circumvent the eye tracking problem or provide an accurate gaze tracking, but at the cost of an atypical interaction. Our solution for estimating the human gaze using head pose tracking is certainly a simplification, however, it makes the interaction with the robot seamless. Moreover, it provides a measurable feedback signal that can be exploited by the robot to adapt on-line its actions to the human behavior. This is a pre-requisite for implementing an on-line robot controller that is capable of taking into account the human (his posture, actions, intent, etc.) when interacting with the robot during a collaborative task. It is also our belief that natural interaction is a requirement for measuring the timing of the human reactions in response to the robot's attention stimuli in a open-ended interaction scenario.

### 4.4. Toward a natural human–robot interaction

In prior work (Ivaldi et al., 2013), we have been investigating how social interaction can influence the learning process of the robot. We showed that social guidance combined with the robot's curiosity could make the robot explore its environment and learn “like a toddler,” focusing on the most informative objects (Oakes and Baumgartner, 2012). The experiments were conducted with biased and unbiased caregivers, proving that even social partners who are not aware of the learning task of the robot were not influencing drastically the performance of the learning process of the robot. The robot's intrinsic motivation system was able to compensate for the different teaching inputs. Those experiments were not completely realistic: the human and the robot were interacting together exchanging objects and toys on a table, which is a natural experimental setting; however, the learning process supposed that the two agents were totally engaged in accomplishing the learning task. In fact this happens quite rarely with participants unfamiliar with the robot, who do not have a perfect understanding of the robot's capabilities and could misinterpret as faults or disengagement, for example, the robot's inactivity periods when its learning system decides what to do and how. In our opinion this is one of the main limitations that prevents performing such experiments with naive participants: it is critical to endow the robot with the social abilities to engage the partner, arouse interest and make interaction as natural as possible, so that everyone could teach to the robot or collaborate with it for doing a task.

With this paper, we made a first attempt in answering how to make the social interaction not only effective for the accomplishment of the task (i.e., teaching something to the robot) but also natural as if the human was teaching to another human. With a simple joint attention mechanism almost all participants perceived the robot as engaged and its behavior quite readable.

A remarkable result that suggest a natural interaction is the average amount of time that the participants in our study spent looking at the robot. According to Argyle and Cook (1976), during human–human interaction people look at each other about 60% of the time, and look more while listening than while talking (during which they give frequent short glances). Interestingly, as shown in Table 3, the participants in our study looked at the robot about 65–67% of the time, which is comparable with the human–human case. This could be a positive indicator of natural interaction^13^.

We showed that the initiating role of the robot in the teaching phase of the task has a consequence on the rhythm of the interaction in the second phase. Intuitively, we were expecting a different perceived engagement in groups HI and RI (that is different ratings in the questionnaire of Table 4), which could reflect the difference in the reaction times of the two groups. The questionnaire results, however, show that there is no significant statistical difference in the evaluation of the quality of interaction in the two cases. While the initiating role of the robot influences the rhythm of interaction, it does not influence the perceived engagement. This means that while the volunteers with a prior experience of the “active” robot react faster in response to a robot's attention utterance, their perception of the quality of the interaction is not different from the one of people with a prior interaction with a “passive” robot. This result could be biased by several factors: the participants' attitude toward robots in general, or to new tasks; their natural attitude when teaching, their engagement toward the task (that was not challenging or motivating for an adult). We plan to perform additional experiments to verify the independence of the engagement with respect to these factors, with more subjects.

Though preliminary, our results provide insights for improving the engagement system of the robot and make interaction with the human more natural and effective. Qualitatively, results indicate that people would certainly prefer the robot to react faster (which is mainly a technological limitation due to the processing time of our modules, but points toward the implementation of anticipatory behaviors); they would also like to see improved behaviors even if they were not necessary for the task accomplishment (more gestures, more natural voice). We plan to perform more experiments to study how introducing these desirable features will change the participant's and observer's perception of the human-likeness of the interaction, particularly their effect on the performance of the robot during learning tasks: could a more natural human–robot interaction yield better robot learning performance? Our intuition is that it could, especially if the people interacting with the robot are naive. Therefore, this work is a first step toward replicating the teaching experiments of Ivaldi et al. (2013) in more ecological and natural conditions with naive people.

Of course, it still remains an open question about how much the iCub's human-like/child-like appearance can influence people's expectations about its behavior. This question was equally raised by Huang and Thomaz (2011) about people interacting with Simon, a Meka humanoid with a child-like head. It is indeed possible that people expectation about the robot performances and movements were disappointed during our interaction experiment, because they were attributing human-like properties and cognitive processes to it, which did not correspond to reality (Stenzel et al., 2012). Hence the negative evaluation of the robot behavior. This is indeed very plausible, because most of the participants were unfamiliar with robots, and not aware of their real capabilities.

### Conflict of interest statement

The authors declare that the research was conducted in the absence of any commercial or financial relationships that could be construed as a potential conflict of interest.

## Footnotes

^1^It has to be remarked that estimating the gaze simply looking at the head movements is quite challenging with respect to estimating the gaze using a combination of head and eyes movements. However, the latter estimation typically requires an external device, such as an eye-tracker, to track the eye movements.

^2^The only criterion for participating to the study was to have never interacted with the robot before—“un-familiarity” or prior non-experience. As the robot is quite famous in the campus, many of them had already seen the robot (for example in photos, flyers or videos) or knew about its existence. Specifically, three in HI and six in RI already knew about it. However, all participants were equally “naive,” as they had never done an experiment with the robot before. Age and sex are not control variables for our study, and interpersonal differences were not relevant.

^3^All the participants were informed, orally by the experimenter and through a printed instruction paper, about the aim of the study, the goal, the measurements, the methodology, and they gave informed consent about participating to the experiment and being filmed for research purposes. The participants were not exposed to any risk of injury, as they were not required to interact physically with the robot.

^4^In the verification phase, the individual trials were (HI) 3, 3, 3, 3, 4, 7, (RI) 4, 5, 5, 5, 5, 7, 9.

^5^Human–human interaction video (only for group A): http://www.youtube.com/watch?v=fUVOFyuGSJM. Human–Robot interaction video (for groups A and B): http://www.youtube.com/watch?v=VrzL7xIB0Nw.

^6^For more details, see http://wiki.icub.org/wiki/UPMC_iCub_project/YARP_ROS_bridge.

^7^Festival: http://www.cstr.ed.ac.uk/projects/festival/. CMU Sphinx: http://cmusphinx.sourceforge.net.

^8^For example, the human could say “The object is orange,” “It is orange,” or simply “Orange.”

^9^As the association color label—color feature is done after the human speech, the human could actually teach a wrong color label to the robot.

^10^This plot is useful to figure the oscillations of the head pitch from the robot head to the table level. An analogous plot for the head yaw can be used to associate each yaw level to the three objects on the table.

^11^In this figure, we report the plots related to head yaw, because the three levels corresponding to the three objects are clearly visible. It is also more interesting with respect to plotting the head pitch, that could only show two levels (the head and table level) and would not allow to distinguish among the three objects, which are all at the same level for the head pitch.

^12^In the experiment, the robot was programmed in a way that it was waiting for the human to match its referential focus. So an erroneous situation—i.e., the human looking at a different object than the one chosen by the robot—would have been caught. However, during the experiments this situation never occurred: the human always looked at the correct object pointed by the robot.

^13^Whether this value is affected by the embodiment induced by the humanoid shape, it is hard to tell: further experiments with different robots are necessary to investigate this question.

## References

[B1] Andry P. Blanchard A. Gaussier P. (2011). Using the rhythm of nonverbal human-robot interaction as a signal for learning. IEEE Trans. Auton. Mental Dev. 3, 30–42 10.1109/TAMD.2010.2097260

[B2] Argyle M. Cook M. (1976). Gaze and Mutual Gaze. Cambridge: Cambridge University Press

[B3] Asada M. Hosoda K. Kuniyoshi Y. Ishiguro H. Inui T. Yoshikawa Y. (2009). Cognitive developmental robotics: a survey. IEEE Trans. Auton. Ment. Dev. 1, 12–34 10.1109/TAMD.2009.2021702

[B4] Biddle B. (1986). Recent developments in role theory. Annu. Rev. Soc. 12, 67–92 10.1146/annurev.so.12.080186.000435

[B5] Boucher J.-D. Pattacini U. Lelong A. Bailly G. Elisei F. Fagel S. (2012). I reach faster when I see you look: gaze effects in human-human and human-robot face-to-face cooperation. Front. Neurorobot. 6, 1–11 10.3389/fnbot.2012.00003 22563315 PMC3342577

[B6] Breazeal C. (2003). Toward social robots. Robot. Auton. Syst. 42, 167–175 10.1016/S0921-8890(02)00373-1

[B7] Breazeal C. Kidd C. D. Thomaz A. L. Hoffman G. Berlin M. (2005). Effects of nonverbal communication on efficiency and robustness in human-robot teamwork, in IEEE/RSJ International Conference on Intelligent Robots and Systems (Cambridge, MA), 383–388 10.1109/IROS.2005.1545011

[B8] Chaminade T. Okka M. M. (2013). Comparing the effect of humanoid and human face for the spatial orientation of attention. Front. Neurorobot. 7:12 10.3389/fnbot.2013.00012 24027525 PMC3759784

[B9] Clark H. (1996). Using Language. Cambridge: Cambridge University Press 10.1017/CBO9780511620539

[B10] Clark H. Wilkes-Gibbs D. (1986). Referring as a collaborative process. Cognition 22, 1–39 10.1016/0010-0277(86)90010-7 3709088

[B11] Cristinacce D. Cootes T. (2006). Feature detection and tracking with constrained local models. Proc. Br. Mach. Vis. Conf. 3, 929–938 10.5244/C.20.95

[B12] Delaherche E. Chetouani M. Bigouret F. Xavier J. Plaza M. Cohen D. (2013). Assessment of the communicative and coordination skills of children with autism spectrum disorders and typically developing children usingsocial signal processing. Res. Autism Spectr. Disord. 7, 741–756 10.1016/j.rasd.2013.02.003

[B13] Delaherche E. Chetouani M. Mahdhaoui A. Saint-Georges C. Viaux S. Cohen D. (2012). Interpersonal synchrony : a survey of evaluation methods across disciplines. IEEE Trans. Affect. Comput. 3, 349–365 10.1109/T-AFFC.2012.12

[B14] Dong W. Lepri B. Pianesi F. Pentland A. (2013). Modeling functional roles dynamics in small group interactions. IEEE Trans. Multimedia 15, 83–95 10.1109/TMM.2012.2225039

[B15] Emery N. (2000). The eyes have it: the neuroethology, function and evolution of social gaze. Neurosci. Biobehav. Rev. 24, 581–604 10.1016/S0149-7634(00)00025-7 10940436

[B16] Fischer K. Lohan K. Saunders J. Nehaniv C. Wrede B. Rohlfing K. (2013). The impact of the contingency of robot feedback on HRI, in International Conference on Collaboration Technologies and Systems (San Diego, CA), 210–217 10.1109/CTS.2013.6567231

[B17] Flanagan J. Rotman G. Reichelt A. Johansson R. (2013). The role of observers' gaze behaviour when watching object manipulation tasks: predicting and evaluating the consequences of action. Phil. Trans. R. Soc. B 368, 20130063 10.1098/rstb.2013.0063 24018725 PMC3758206

[B18] Frith C. (2012). The role of metacognition in human social interactions. Phil. Trans. R. Soc. B 367, 2213–2223 10.1098/rstb.2012.0123 22734064 PMC3385688

[B19] Georgiou I. Becchio C. Glover S. Castiello U. (2007). Different action patterns for cooperative and competitive behaviour. Cognition 102, 415–433 10.1016/j.cognition.2006.01.008 16516188

[B20] Huang C.-M. Thomaz A. L. (2011). Effects of responding to, initiating and ensuring joint attention in human-robot interaction, in Proceedings of IEEE RO-MAN (Atlanta, GA), 65–71 10.1109/ROMAN.2011.6005230

[B21] Hung H. Huang Y. Friedland G. Gatica-Perez D. (2008). Estimating the dominant person in multi-party conversations using speaker diarization strategies, in IEEE International Conference on Acoustics, Speech and Signal Processing (Las Vegas, NV), 2197–2200 10.1109/ICASSP.2008.4518080

[B22] Ivaldi S. Lyubova N. Gérardeaux-Viret D. Droniou A. Anzalone S. M. Chetouani M. (2012). Perception and human interaction for developmental learning of objects and affordances, in Proceedings of IEEE-RAS International Conference on Humanoid Robots (Osaka, Japan). 10.1109/HUMANOIDS.2012.6651528

[B23] Ivaldi S. Nguyen S. M. Lyubova N. Droniou A. Padois V. Filliat D. (2013). Object learning through active exploration. IEEE Trans. Auton. Ment. Dev. 1–18 10.1109/TAMD.2013.2280614

[B24] Jarrassé N. Sanguinetti V. Burdet E. (2013). Slaves no longer: review on role assignment for human-robot joint motor action. Adapt. Behav. (Sage) 1–13 10.1177/1059712313481044

[B25] Kaplan F. Hafner V. V. (2006). The challenges of joint attention. Interact. Stud. 7, 135–169 10.1075/is.7.2.04kap

[B26] Knoblich G. Butterfill S. Sebanz N. (2011). Psychological research on joint action: theory and data, in The Psychology of Learning and Motivation, Vol. 54, ed B. Ross (Burlington: Academic Press), 59–101

[B27] Meltzoff A. N. (2007). 'Like me': a foundation for social cognition. Dev. Sci. 10, 126–134 10.1111/j.1467-7687.2007.00574.x 17181710 PMC1852489

[B28] Moll H. Meltzoff A. N. (2011). Perspective-taking and its foundation in joint attention, in Perception, Causation, and Objectivity. Issues in Philosophy and Psychology, eds Eilan N. Lerman H. Roessler J. (Oxford: Oxford University Press), 286–304 10.1093/acprof:oso/9780199692040.003.0016

[B29] Morales M. Mundy P. Rojas J. (1998). Following the direction of gaze and language development in 6-month-olds. Infant Behav. Dev. 21, 373–377 10.1016/S0163-6383(98)90014-5

[B30] Natale L. Nori F. Metta G. Fumagalli M. Ivaldi S. Pattacini U. (2013). The iCub platform: a tool for studying intrinsically motivated learning, in Intrinsically Motivated Learning in Natural and Artificial Systems, eds Baldassarre G. Mirolli M. (Berlin: Springer-Verlag), 433–458 10.1007/978-3-642-32375-1

[B31] Oakes L. M. Baumgartner H. A. (2012). Manual object exploration and learning about object features in human infants, in IEEE International Conference on Development and Learning, ICDL-EPIROB (San Diego, CA). 10.1109/DevLrn.2012.6400819

[B32] Obhi S. S. Sebanz N. (2011). Moving together: toward understanding the mechanisms of joint action. Exp. Brain Res. 211, 329–336 10.1007/s00221-011-2721-0 21573952

[B33] Pattacini U. Nori F. Natale L. Metta G. Sandini G. (2010). An experimental evaluation of a novel minimum-jerk cartesian controller for humanoid robots, in IEEE/RSJ International Conference on Intelligent Robots and Systems (Taipei), 1668–1674 10.1109/IROS.2010.5650851

[B34] Piaget J. (1962). Play, Dreams and Imitation in Childhood. Cambridge, MA: MIT Press

[B35] Posner M. I. (1980). Orienting of attention. Q. J. Exp. Psychol. 32, 3–25 10.1080/00335558008248231 7367577

[B36] Reed K. Patton J. Peshkin M. (2007). Replicating human-human physical interaction, in 2007 IEEE International Conference on Robotics and Automation (Roma), 3615–3620 10.1109/ROBOT.2007.364032

[B37] Rich C. Ponsler B. Holroyd A. Sidner C. L. (2010). Recognizing engagement in human-robot interaction, in Proceedings of ACM/IEEE International Conference on Human-Robot Interaction (HRI) (Osaka), 375–382 10.1109/HRI.2010.5453163

[B38] Rousseau W. Anzalone S. M. Chetouani M. Sigaud O. Ivaldi S. (2013). Learning object names through shared attention. in International Conference on Intelligents Robots and Systems - Workshop on Developmental Social Robotics. (Tokyo).

[B39] Sacks H. Schegloff E. A. Jefferson G. (1974). A simplest systematics for the organization of turn-taking for conversation. Language 50, 696–735 10.2307/412243

[B40] Sciutti A. Bisio A. Nori F. Metta G. Fadiga L. Sandini G. (2012). Anticipatory gaze in human-robot interactions, in Gaze in HRI from Modeling to Communication, Workshop at 7th ACM/IEEE International Conference on HRI. (Boston, MA).

[B41] Sebanz N. Bekkering H. Knoblich G. (2006). Joint action: bodies and minds moving together. Trends Cogn. Sci. 10, 70–76 10.1016/j.tics.2005.12.009 16406326

[B42] Shotton J. Fitzgibbon A. Cook M. Sharp T. Finocchio M. Moore R. (2011). Real-time human pose recognition in parts from single depth images, in IEEE CVPR (Providence, RI). 10.1109/CVPR.2011.5995316

[B43] Skantze G. Hjalmarsson A. Oertel C. (2013). Exploring the effects of gaze and pauses in situated human-robot interaction, in Proceedings of the 14th Annual Meeting of Special Interest Group on Doscourse and Dialogue - SIGDial, (Metz), 375–383

[B44] Smith L. Gasser M. (2005). The development of embodied cognition: six lessons from babies. Artif. Life 11, 13–29 10.1162/1064546053278973 15811218

[B45] Staudte M. Crocker M. W. (2010). The dynamics of referential speaker gaze: order is important, synchronization, not so much, in Workshop on Modeling Human Communication Dynamics—NIPS. (Vancouver, BC).

[B46] Staudte M. Crocker M. W. (2011). Investigating joint attention mechanisms through spoken human-robot interaction. Cognition 120, 268–291 10.1016/j.cognition.2011.05.005 21665198

[B47] Stefanov N. Peer A. Buss M. (2009). Role determination in human-human interaction, in 3rd Joint EuroHaptics Conference and World Haptics, (Salt Lake City, UT) 51–56 10.1109/WHC.2009.4810846

[B48] Steinfeld A. Fong T. Kaber D. Lewis M. Scholtz J. Schultz A. (2006). Common metrics for human-robot interaction, in ACM SIGCHI/SIGART Conference on Human-Robot Interaction (Salt Lake City, UT), 33–40 10.1145/1121241.1121249

[B49] Stenzel A. Bou M. A. T. Chinellato E. del Pobil A. P. Lappe M. Liepelt R. (2012). When humanoid robots become like human-like interaction partners: corepresentation of robotic actions. J. Exp. Psychol. Hum. Percept. Perform. 38, 1073–1077 10.1037/a0029493 22866762

[B50] Sumioka H. Hosoda K. Yoshikawa Y. Asada M. (2007). Acquisition of joint attention through natural interaction utilizing motion cues. Adv. Robot. 21, 983–999 10.1163/156855307781035637

[B51] Sumioka H. Yoshikawa Y. Asada M. (2010). Reproducing interaction contingency toward open-ended development of social actions: case study on joint attention. IEEE Trans. Auton. Ment. Dev. 2, 40–50 10.1109/TAMD.2010.2042167

[B52] Tanenhaus M. K. Spivey-Knowlton M. J. Eberhard K. M. Sedivy J. C. (1995). Integration of visual and linguistic information in spoken language comprehension. Science 268, 1632–1634 10.1126/science.7777863 7777863

[B53] ter Maat M. Truong K. P. Heylen D. (2010). How turn-taking strategies influence users' impressions of an agent, in Intelligent Virtual Agents. Lecture Notes in Computer Science, Vol. 6356, eds Allbeck J. Badler N. Bickmore T. Pelachaud C. Safonova A. (Berlin: Springer), 441–453

[B54] ter Maat M. Truong K. P. Heylen D. (2011). How agents' turn-taking strategies influence impressions and response behaviors. Presence 20, 412–430 10.1162/PRES_a_00064

[B55] Thomaz A. Chao C. (2011). Turn-taking based on information flow for fluent human-robot interaction. AI Mag. Spec. Issue Dialog Robot. 32, 53–63 Available online at: http://www.aaai.org/ojs/index.php/aimagazine/article/view/2379

[B56] Tomasello M. (2009). Why We Cooperate. Vol. 206 Cambridge: MIT press

[B57] Tomasello M. Carpenter M. Call M. Behne J. T. Moll H. (2005). Understanding and sharing intentions: the origin of cultural cognition. Behav. Brain Sci. 28, 675–691 10.1017/S0140525X05000129 16262930

[B58] Ugur E. Celikkanat H. Sahin E. Nagai Y. Oztop E. (2011). Learning to grasp with parental scaffolding, in IEEE International Conference on Humanoid Robotics (Bled), 480–486 10.1109/Humanoids.2011.6100890

[B59] Wilcox R. Nikolaidis S. Shah J. A. (2012). Optimization of temporal dynamics for adaptive human-Robot interaction in assembly manufacturing, in Robotics Science and Systems VIII eds Roy N. Newman P. Srinivasa S. (Cambridge, MA: MIT Press), 441–448

[B60] Wiltermuth S. S. Heath C. (2009). Synchrony and cooperation. Psychol. Sci. 20, 1–5 10.1111/j.1467-9280.2008.02253.x 19152536

[B61] Yamazaki A. Yamazaki K. Kuno Y. Burdelski M. Kawashima M. Kuzuoka H. (2008). Precision timing in human-robot interaction: coordination of head movement and utterance, in Proceedings of the SIGCHI Conference on Human Factors in Computing Systems (New York, NY: ACM), 131–140 10.1145/1357054.1357077

[B62] Yu C. Scheutz M. Schermerhorn P. (2010). Investigating multimodal real-time patterns of joint attention in an hri word learning task, in ACM/IEEE International Conference on Human-Robot Interaction (HRI) (Osaka), 309–316 10.1109/HRI.2010.5453181

